# Effect of Chronic Administration of Nickel on Affective and Cognitive Behavior in Male and Female Rats: Possible Implication of Oxidative Stress Pathway

**DOI:** 10.3390/brainsci8080141

**Published:** 2018-07-31

**Authors:** Mouloud Lamtai, Jihane Chaibat, Sihame Ouakki, Oussama Zghari, Abdelhalem Mesfioui, Aboubaker El Hessni, El-Housseine Rifi, Ilias Marmouzi, Azzouz Essamri, Ali Ouichou

**Affiliations:** 1 Unit of Nervous and Endocrine Physiology, Laboratory of Genetics, Neuroendocrinology and Biotechnology, Faculty of Science, University Ibn Tofail, Kenitra 14000, Morocco; jihanechaibat@gmail.com (J.C.); ouichouali@yahoo.fr (S.O.); zgharioussama91@gmail.com (O.Z.); a.mesfioui@yahoo.fr (A.M.); elhessni70@yahoo.fr (A.E.H.); ouichou@hotmail.com (A.O.); 2 Laboratory of Synthesis Organic and Extraction Processes, Department of Chemistry, Faculty of Science, University Ibn Tofail, Kenitra 14000, Morocco; elhosseinr@yahoo.fr; 3 Laboratoire de Pharmacologie et Toxicologie, équipe de Pharmacocinétique, Faculté de Médecine et de Pharmacie, University Mohammed V in Rabat, BP 6203, Rabat Instituts, Rabat 10100, Morocco; ilias.marmouzi@gmail.com; 4 Laboratory of Agro-Resources and Process Engineering, Faculty of Science, University Ibn Tofail, Kenitra 14000, Morocco; essamri@gmail.com

**Keywords:** nickel, depression-like, anxiety-like, memory, oxidative stress

## Abstract

Nickel (Ni) toxicity has been reported to produce biochemical and behavioral dysfunction. The present study was undertaken to examine whether Ni chronic administration can induce alterations of affective and cognitive behavior and oxidative stress in male and female rats. Twenty-four rats, for each gender, divided into control and three test groups (*n* = 6), were injected intraperitoneally with saline (0.9% NaCl) or NiCl2 (0.25 mg/kg, 0.5 mg/kg and 1 mg/kg) for 8 weeks. After treatment period, animals were tested in the open-field, elevated plus maze tests for anxiety-like behavior, and forced swimming test for depression-like behavior. The Morris Water Maze was used to evaluate the spatial learning and memory. The hippocampus of each animal was taken for biochemical examination. The results showed that Ni administration dose dependently increased anxiety-like behavior in both tests. A significant increase in depression-like symptoms was also exhibited by Ni treated rats. In the Morris Water Maze test, the spatial learning and memory were significantly impaired just in males treated with 1 mg/kg of Ni. With regard to biochemical analysis, activity of catalase (CAT) and superoxide dismutase (SOD) were significantly decreased, while the levels of nitric oxide (NO) and lipid peroxidation (LPO) in the hippocampus were significantly increased in the Ni-treated groups. Consequently, chronic Ni administration induced behavioral and biochemical dysfunctions.

## 1. Introduction

It is well known that nickel (Ni) is an essential element for human and several animal species [1,2]. Imbalanced Ni homeostasis either by deficiency or by overload of this metal is associated with organ dysfunction that leads to various physiological and behavioral disorders. Ni deficiency inhibits growth, reduces reproductive rate, and alters glucose and lipid metabolism, which are associated with anemia, alternations of other metal ion contents, and reduced activity of several enzymes in animals [3]. In contrast, continuous exposure to high levels of Ni leads to multiple toxic effects in various organs, including the lungs, liver, kidneys, and brain [4,5,6].

The nervous system is one of the systems affected by Ni toxicity [7]. It may be taken up into the brain through failures of the blood–brain barrier (BBB), and also via the olfactory pathway [8,9,10], and then accumulates in the cerebral cortex and whole brain [8,11], leading to a cytotoxicity in different types of nerve cells [7,11,12]; a variety of neurological symptoms such as headaches, giddiness, tiredness, lethargy, and ataxia [6]; apoptosis of olfactory sensory and cerebral cortex neurons and behavioral deficiencies; and disrupts neurotransmitters [13,14,15,16]. 

It is known that changes in neurochemistry often correlate with behavioral disturbance [17]. Animal studies have shown that Ni exposure leads to increased aggressive behavior and affective disorders, and impaired memory processes and exploring activity [11,18]. It has been also demonstrated that Ni has a neuromodulatory role; it can interfere with acetylcholine release from peripheral nerve terminals in vitro [19] and decrease dopamine, norepinephrine, and serotonin levels in certain rat brain regions and change the gene expression of the glutamate receptors [7,16,20,21]. 

Among the many mechanisms implicated in nickel-mediated neurotoxicity, oxidative stress has been proposed to play a central role; it can damage tissue, including central nervous system (CNS), leading to impaired neuronal function and alteration in the physicochemical properties of cell membranes, and eventually disrupt the vital functions and overall brain activity [6,22]. As a result, it can alter neurotransmission [23]. Importantly, oxidative stress reduces gamma-Aminobutyric acid (GABA) levels [24] and alters GABA uptake [25]. Certain diseases associated with oxidative stress disturbances such as neurodegenerative diseases like Alzheimer’s and Parkinson’s Diseases [26,27], and neuropsychiatric diseases including schizophrenia and some forms of behavior, such as aggressiveness, depression, and anxiety, and also to deterioration of short-term spatial memory [23,28,29,30,31].

To our knowledge, no literature is available to address the effect of Ni on depression-like behavior, anxiety-like behavior, learning, and memory in rats in association with oxidative stress. So, the present study was designed to determine the effects of chronic administration of Ni on animal affective and cognitive behavior and on oxidative stress levels in male and female rats.

## 2. Material & Methods

### 2.1. Animals and Experimental Conditions

This study was performed on male and female Wistar rats initially weighing (120 ± 20 g). Animals, from breeding from the Faculty of Life Sciences, University Ibn Tofaïl, were grouped into six rats in each cage (36 cm long, 20 cm wide, and 15 cm high). The space reserved for the breeding and the survival of the rats respects the well-being of the animals. All rats were maintained under LD 12/12 (12 h Light/12 h Darkness) and at a standard temperature (21 ± 1 °C). Water and food were provided ad libitum. All experimental procedures were approved by the University Ethics Committee for Animal Experiments.

The rats are divided into four groups of six animals each (six males and six females). For each sex, the different groups of rats are distributed as follows:

- 1st group: rats control receiving daily an intraperitoneal injection of Nacl 0.9%.
- 2nd group: rats receiving daily a dose of 0.25 mg/kg of Ni.
- 3rd group: rats receiving daily a dose of 0.5 mg/kg of Ni.
- 4th group: rats receiving daily 1 mg/kg of Ni.

Saline solution or NiCl_2_ (obtained from Sigma-Aldrich, St. Louis, MO, USA), used in the present work was injected intraperitoneally and chronically at the rate of one injection per day for 8 weeks. All injections were carried out between 4:00 pm and 4:30 pm. In nature, Ni exists in very small quantities, even in the form of traces. For this reason, we have the originality to use doses even lower than those reported in the literature, namely 0.25 and 0.5 mg/kg, in order to mimic or approach the natural doses of metal.

### 2.2. Neurobehavioral Tests

Twenty-four hours after the end of the 8 weeks corresponding to the treatment period, the animals were submitted to behavioral tests at the rate of one test per day, in the following order: the Open field test (OFT), followed by the Elevated plus maze (EPM), Forced swimming test (FST), and Morris water maze test (MWM), respectively [32,33,34]. The tests were performed between 8 am and 12 am.

### 2.3. Anxiety-Like Measurement 

***Open Field Test*:** The OFT is used to measure the anxiety-like behavior in rodents [35,36]. The maze adopted is made of wood (100 cm × 100 cm) enclosed with 40 cm high walls and placed under strong illumination (100 W, 2 m above the apparatus). The area was divided into 25 squares (20 cm × 20 cm), defined as 9 central and 16 peripheral squares. At the beginning of the 10-min test, the animal was placed in the center of the apparatus and its behavior was videotaped for subsequent analysis. The quantified parameters were the time spent in the center of the area (TCA) and the number of returns to the center (NRC). Central perimeter residence time is used as a measure of anxiety. The number of returns to the central area is also an indicator of the emotional reactivity. The central area of a novel environment is anxiogenic and aversive, and the behavioral inhibition appears therefore as an avoidance behavior towards the central zone of the OFT. Locomotors activity is represented by number of total squares (NTS). The apparatus was cleaned between each examination using 70% ethyl alcohol.

***Test of the Elevated Plus Maze (EPM)*:** The EPM is an ethological model of anxiety in rodents provoked by the novelty and repulsion as a result of elevation and illumination of the maze [37,38]. This test is based on the creation of a conflict between the exploratory drive of the rat and its innate fear of open and exposed areas; it has been validated for the detection of emotional responses to anxiogenic and anxiolytic substances. Thus, increased open-arms exploration indicates reduced anxiety-related behavior. The EPM consists of a wooden plus-shaped platform elevated 70 cm above the floor. Two of the opposing arms (50 cm × 10 cm) are closed by 40 cm high side and end walls, having an open roof. In order to avoid fall, the other two arms (open arms) were surrounded by 0.5 cm high edge, the four arms had at their intersection a central platform (10 cm × 10 cm). A 100-W lamp was placed exactly over the central platform. At the beginning of the test, the rats were placed on the central area of the maze facing an open arm. The following parameters of anxiety-related behavior were measured during the 5-min testing period: (1) entries into open arms (EOA), (2) time spent on the open arms (TOA), (3) and number of full entries into the arms (TAE). Decreased anxiety-like behavior is illustrated by a significant statistical increase of parameters in open arms (time, entries, or both). The total number of the entries into all arms provides general hyperactivity. To eliminate any lingering olfactory cues, the apparatus was cleaned between each examination using 70% ethyl alcohol. 

***Depression-Like Measurement in Forced Swimming Test (FST):*** The FST is an excellent maze used to assess the depressive-like behavior [39]. Swimming sessions were conducted by placing the rat in individual glass cylinders (height = 50 cm; diameter = 30 cm) containing 30 cm of water at (23 ± 2 °C). During the session, rats were forced to swim for 5 min and the duration of immobility was measured. The latency to the first bout of immobility was also recorded starting immediately after placing the rats in the cylinder. A rat was judged immobile when it ceased all active behaviors (i.e., struggling, swimming, and jumping) and remained passively floating or making minimal movements necessary to maintain the nostrils above water. High percent time floating is interpreted as an increased depressive-like response [39,40].

### 2.4. Cognitive Measurement

***Morris Water Maze Test*:** The water maze task employed was an adaptation of the hidden escape paradigm described by Morris [41,42]. A platform (30 cm high, 10 cm in diameter) was placed in an off-center position inside a circular grey acrylic tank (110 cm in diameter and 50 cm high) containing warm water (24 °C) filled to a level 1 cm above the platform, making it invisible. To ensure the platform was hidden the water was made opaque using a small amount of black non-toxic water-based poster paint. The water maze was located in a room (5.2 m × 2.4 m) with posters and furniture around the walls, which served as extra-maze visual cues. During testing, the room was dimly lit with diffuse white light (30 lx). The paradigm consisted of four trials per day over four consecutive days in which each rat was removed from its holding cage using a plastic container and released from a randomly assigned start location (East, North, South, or West). If the rat did not find the platform within 60 s, it was guided to the platform and allowed to stay on it for approximately 10 s. The latency to locate the hidden platform was recorded for each trial as a measure of spatial learning performance. On the fifth day, the rats were given a single 60 s probe trial in which the platform was removed. The quadrant in which the platform was previously situated was defined as the target quadrant. The percentage time spent in the target quadrant was analysed as a measure of spatial memory performance.

### 2.5. Biochemical Examination

One day after the end of the behavioral tests, all animals were firstly anesthetized and then sacrificed by decapitation. Brains were quickly removed and maintained at low temperature on ice. The hippocampus was rapidly and gently removed and separated from surrounding tissues and homogenized in phosphate buffer at PH: 7.4 (*w*/*v*), centrifuged at 1500 rpm for 10 min, and the resulting supernatant was used in the biochemical assays [33].

***Lipid peroxidation assay:*** The formation of lipid peroxides during lipid peroxidation process was analysed by measuring the thiobarbituric-acid-reacting substances (TBARS) in cells, as previously described by Draper and Hadley, (1990) [43]. Briefly, the samples were mixed with 1 mL of trichloroacetic acid 10% and 1 mL of thiobarbituric acid 0.67%, then heated in a boiling water bath for 15 min, and butanol (2:1 *v*/*v*) was added to the solution. After centrifugation (800 g/5 min), the TBARS were determined by the absorbance at 535 nm [44]. 

***Nitrite/nitrate assay:*** In biological systems, conversion of nitric oxide (NO) in aqueous solution to nitrite and nitrate is thought to favour nitrite production. It has been reported that nitrite is the only stable end-product of the autooxidation of NO in aqueous solution and measurement of nitrite concentrations in the serum and tissue homogenates is widely accepted as an index of NO synthase (NOS) activity [45].

Therefore, concentrations of nitrite in tissue homogenates were measured by using the diazotization method based on the Griess reaction, which is an indirect assay for NO production [46]. Briefly, samples (500 µL) were pipetted into tubes and an equal volume of Griess reagent (1% sulphanylamide (1 mL) and 0.1% *N*-1-naphtylethylenediamine dihydrochloride (1 mL) in 2.5% ortophosphoric acid) was added to each tube. After incubation for 30 min at room temperature, absorbance was measured at 540 nm. Linear regression analysis was used to calculate the nitrite concentrations in the serum and the tissue homogenates from the standard calibration curves of sodium nitrite. tissue nitrite levels were expressed as µmol/g of tissue.

***Determination of Superoxide Dismutase (SOD) activity*:** The superoxide dismutase (SOD) activity was determined according to the method described by Beauchamp and Fridovich (1971) [47]. The principle of SOD activity assay was based on the inhibition of nitroblue tetrazolium (NBT) reduction. Illumination of riboflavin in the presence of O_2_ and electron donor like methionine generates superoxide anions and this has been used as the basis of assay of SOD. The 1 mL reaction mixture consisted of 0.94 mL 50 mM phosphate buffer (pH 7.4) containing 12 mM methionine, 75 μM NBT, 0.1 mM EDTA, 0.025% Triton X-100 and 2 μM riboflavin, and 0.06 mL Supernatant. The assay was carried out by placing the test-tubes in yellow light for 10 min. Control without the enzyme source was always included. The reduction of NBT by superoxide radicals to blue-coloured formazan was followed at 560 nm. “One unit of SOD activity is defined as that amount of enzyme required to inhibit the reduction of NBT by 50% under the specified conditions”. The specific activity was expressed as U/g of hippocampal tissue.

***Determination of Catalase (CAT) activity*:** CAT activity was measured by the method of Aebi (1984) [48]. Supernatant (50 μL) was added to cuvette containing 1.95 mL of 50 mM phosphate buffer (pH 7.4). Reaction was started by the addition of 1.0 mL of freshly prepared 50 mM H_2_O_2_. The rate of decomposition of H_2_O_2_ was measured spectrophotometrically from changes in absorbance at 240 nm. Activity of catalase was expressed as international units (I.U)/g of tissue (i.e., in μmol H_2_O_2_ destroyed/min/g of tissue, at 25 °C).

### 2.6. Statistical Analysis 

Behavioral data and biochemical parameters were analyzed by two-way ANOVA using SPSS (version 22 SPSS, Chicago, IL, USA). Post hoc comparisons were made using the Tukey’s test. ANOVA repeat measures were used for the Morris water maze test. All data are expressed as the means ± standard error of the means (S.E.M.). In order to estimate the gender effect, we expressed the results for each sex as a percentage of the basal level (% BL) represented by the respective control and considered as being 100%. Differences were considered significant when *p* < 0.05, very significant when *p* < 0.01 and highly significant when *p* < 0.001.

## 3. Results

### 3.1. Effect of Nickel on the Levels of Anxiety-Like Measured in the OFT

#### 3.1.1. Time Spent in the Central Area (TCA)

**Figure 1:**
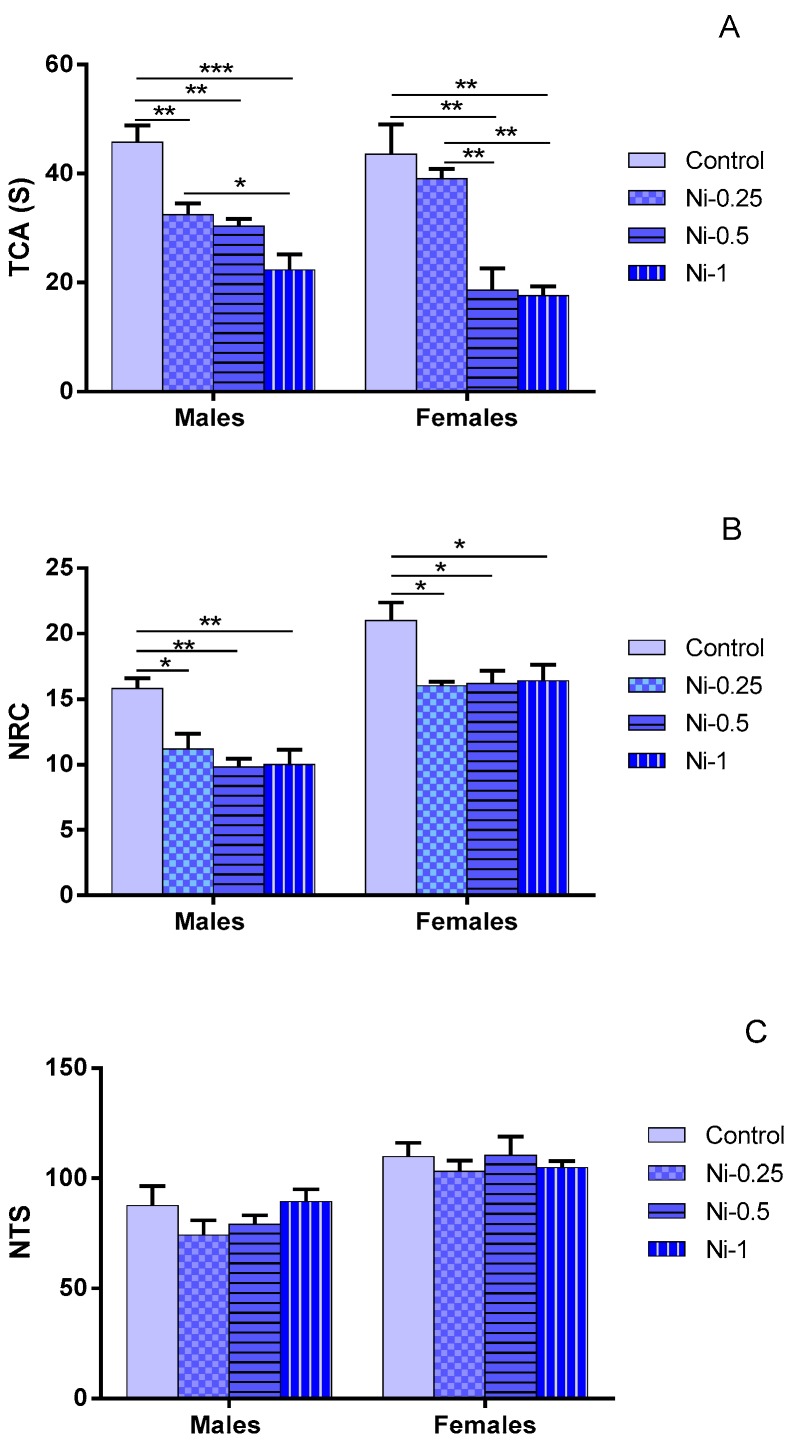
(**A**) Total amount time spent in the center (TCA); (**B**) Number of return into center area of the arena in the open-field behavior apparatus (NRC); and (**C**) Number of total squares (NTS) in the open field in female and male rats after 2 month of treatment with 0.9% of NaCl (Control), 0.25 mg/kg (Ni-0.25), 0.5 mg/kg (Ni-0.5), and 1 mg/Kg (Ni-1) of Ni. Results are expressed as mean ± SEM. The significance level is 0.05. * *p* < 0.05, ** *p* < 0.01, *** *p* < 0.001.

This parameter was affected by Ni treatment (F_(3.32)_ = 26.53, *p* < 0.001) (Figure 1A). In males, Ni affects TCA in dose-dependent manner at doses of 0.25, 0.5 and 1 mg/kg since it decreases the TCA in comparison with the control group (Cont/Ni-0.25: *p* < 0.01, Cont/Ni-0.5: *p* < 0.01 and Cont/Ni-1: *p* < 0.001 respectively). Ni induced mean average decrease of 29%, 34%, and 51% at doses of 0.25, 0.5, and 1 mg/kg respectively. In addition, there is a statistically significant difference between the groups Ni-0.25/Ni-1 (*p* < 0.05). In contrast, no difference was noted between Ni-0.25/Ni-0.5 and Ni-0.5/Ni-1 (*p* > 0.05).

In females, at doses of 0.5 and 1 mg/kg, Ni decreases significantly the TCA in comparison with control group (*p* < 0.01 and *p* < 0.01), while at dose of 0.25 mg/kg this metal did not induce any significant change in this parameter (*p* > 0.05). It induced mean average decrease of 11%, 57%, and 59% at doses of 0.25, 0.5, and 1 mg/kg respectively. Indeed, there is a statistically significant difference between the groups Ni-0.25/Ni-0.5 and Ni-0.25/Ni-1 (*p* < 0.01). In contrast, no difference was noted between Ni-0.5/Ni-1 groups (*p* > 0.05).

#### 3.1.2. Number of Returns to the Center (NRC)

The treatment factor significantly affected the NRC (F_(3.32)_ = 13.10, *p* < 0.001). The effect of Ni is observed in males with referring to NRC parameter (Figure 1B). At doses of 0.25, 0.5, and 1 mg/kg, Ni significantly reduced the NRC compared with the control group (Cont/Ni-0.25: *p* < 0.05, Cont/Ni-0.5: *p* < 0.01, and Cont/Ni-1: *p* < 0.01, respectively). Ni induced mean average decrease of 29%, 38%, and 37% at doses of 0.25, 0.5, and 1 mg/kg respectively.

In females also, Ni decrease significantly NRC in all treated group in comparison with the control group (Cont/Ni-0.25: *p* < 0.05, Cont/Ni-0.5: *p* < 0.05, and Cont/Ni-1: *p* < 0.05, respectively). It induced mean average decrease of 27%, 28%, and 27% at doses of 0.25, 0.5, and 1 mg/kg respectively. In both sexes, no statistically significant difference was observed with comparing different treated Ni groups (*p* > 0.05).

#### 3.1.3. Number of Total Squares (NTS)

Locomotors activity was unaffected by any treatment (F_(3.32)_ = 30.49, *p* > 0.05), and no effect of sex (F_(1.32)_ = 1.49, *p* > 0.05) was noted (Figure 1C). The values of all groups were comparable. Even though it is not significant, we observed a slight increase in the total activity of females compared to males.

### 3.2. Effect of Ni on Anxiety Levels Measured in Elevated Plus Maze Test (EPM)

#### 3.2.1. Time Spent in Open Arms (TOA)

**Figure 2:**
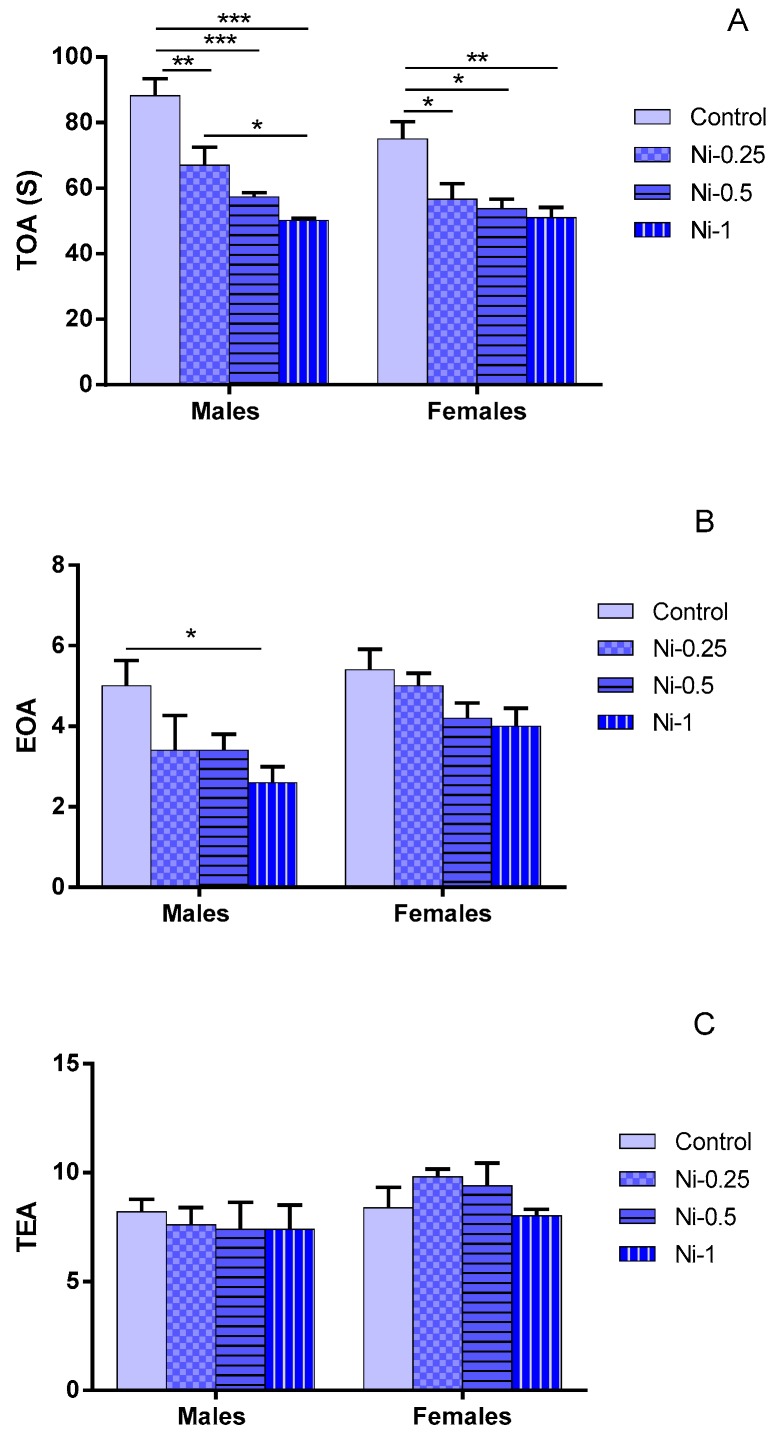
(**A**) Number of entries in exposed arms (EOA); (**B**) Total amount of time spent in exposed arms (TOA); and (**C**) Total number of arms entries (TEA) in elevated plus maze in female and male rats after 2 month of treatment with 0.9% of NaCl (Control), 0.25 mg/kg (Ni-0.25), 0.5 mg/kg (Ni-0.5), and 1 mg/Kg (Ni-1) of Ni. Results are expressed as mean ± SEM. The significance level is 0.05. * *p* < 0.05, ** *p* < 0.01, *** *p* < 0.001.

Statistical analysis showed that TOA was significantly affected by the Ni treatment factor (F_(3.32)_ = 22.9, *p* < 0.001) (Figure 2A). In males, Ni affects TOA in dose-dependent manner, between 0.25 and 1 mg/kg it decreases the TOA in comparison with the control group (Cont/Ni-0.25: *p* < 0.01, Cont/Ni-0.5: *p* < 0.001, and Cont/Ni-1: *p* < 0.001, respectively). Ni induced mean average decrease of 24%, 34%, and 43% at doses of 0.25, 0.5, and 1 mg/kg respectively. Indeed, there is a statistically significant difference between the groups Ni-0.25/Ni-1 (*p* < 0.05). In contrast, no difference was noted between Ni-0.25/Ni-0.5 and Ni-0.5/Ni-1 (*p* > 0.05).

In females, Ni affects TOA at doses of 0.25, 0.5 and 1 mg/kg since it decreases the TOA in comparison with the control group (Cont/Ni-0.25: *p* < 0.05, Cont/Ni-0.5: *p* < 0.05, and Cont/Ni-1: *p* < 0.01, respectively). It induced mean average decrease of 25%, 28%, and 32% at doses of 0.25, 0.5, and 1 mg/kg respectively. No statistically significant difference was observed with comparing different treated Ni groups (*p* > 0.05). 

#### 3.2.2. Entry to Open Arms (EOA)

This parameter was affected by Ni treatment (F_(3.32)_ = 4.76, *p* < 0.01) (Figure 2B). In males, at dose of 1 mg/kg, Ni decreases significantly the EOA in comparison with control group (*p* < 0.05), while at doses of 0.25 and 0.5 mg/kg this metal did not induce any significant change in this parameter (*p* > 0.05). 

In females also, Ni did not induce any significant change in this parameter (*p* > 0.05). Indeed, in both sexes, no statistically significant difference was observed with comparing different treated Ni groups (*p* > 0.05).

Similar results were observed when considering the relative comparison (EOA % BL) between treated Ni and control groups. Thus, Ni induced mean average decrease of 32%, 32%, and 48% in males, and of 7%, 22%, and 26% in females respectively, at doses of 0.25, 0.5, and 1 mg/kg respectively. 

#### 3.2.3. Total Entries in Arms (TEA) 

In contrast to TOA and EOA parameters, Ni was no significant effect on locomotors activity (TEA) represented (Figure 2C), whatever the dose considered (*p* > 0.05). 

### 3.3. Effect of Nickel on Depressive-Like Performances Measured by Forced Swimming Test (FST)

#### 3.3.1. Immobility Time (TIM) 

**Figure 3:**
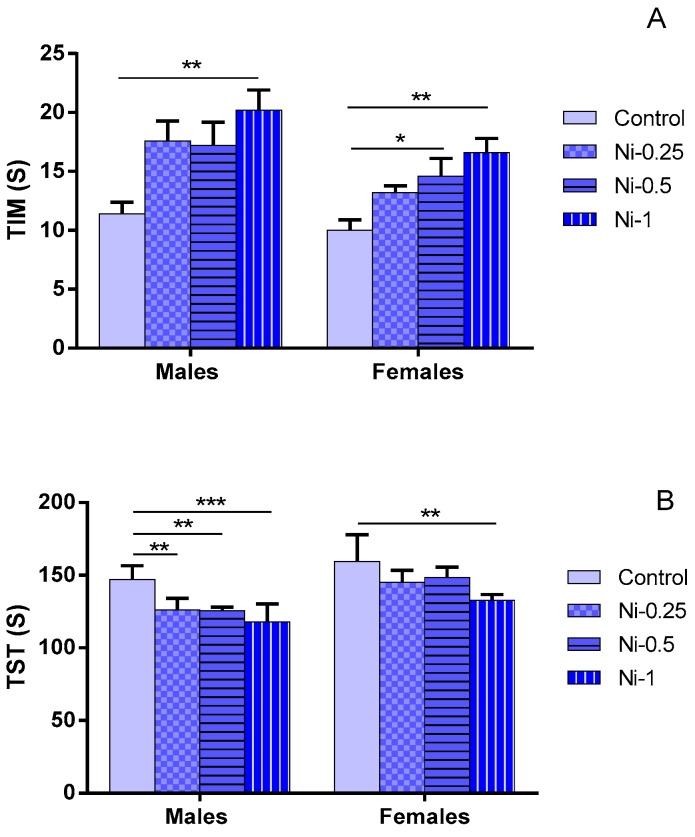
(**A**) Immobility time expressed in seconds (s) (TIM); (**B**) Struggling time (STS) in Forced swimming test expressed in seconds (s) in female and male rats after 2 months of treatment with 0.9% of NaCl (Control), 0.25 mg/kg (Ni-0.25), 0.5 mg/kg (Ni-0.5), and 1 mg/Kg (Ni-1) of Ni. Results are represented as mean ± SEM. The significance level is 0.05. * *p* < 0.05, ** *p* < 0.01, *** *p* < 0.001.

Statistical analysis showed that TIM was significantly affected by the Ni treatment (F_(3.32)_ = 10.04, *p* < 0.001) (Figure 3A). In males, at dose of 1 mg/kg, Ni increased significantly TIM in comparison with control group (*p* < 0.01), while at doses of 0.25 and 0.5 mg/kg this metal did not induce any significant change in this parameter (*p* > 0.05). It induced mean average increase of 54%, 51%, and 77% at doses of 0.25, 0.5, and 1 mg/kg respectively.

In females, at doses of 0.5 and 1 mg/kg, Ni increases significantly the TIM in comparison with control group (*p* < 0.05 and *p* < 0.01 respectively), while at dose of 0.25 mg/kg this metal did not induce any significant change in this parameter (*p* > 0.05). The metal induced mean average increase of 32%, 46%, and 66% at doses of 0.25, 0.5, and 1 mg/kg respectively. In addition, in both sexes, no statistically significant difference was observed with comparing different treated Ni groups (*p* > 0.05).

#### 3.3.2. Struggling Time (TST) 

Statistical analysis showed that TST was significantly affected by the Ni treatment (F_(3.32)_ = 13.11, *p* < 0.001). The dose-dependent effect of Ni is observed in males with referring to TST parameter (Figure 3B). At doses of 0.25, 0.5, and 1 mg/kg, Ni decreases the TST compared with the control group (*p* < 0.01, *p* < 0.01, and *p* < 0.001 respectively).

In females, at dose of 1 mg/kg, Ni decreases significantly TST in comparison with control group (*p* < 0.01), while at doses of 0.25 and 0.5 mg/kg this metal did not induce any significant change in this parameter (*p* > 0.05). Indeed, in both sexes, no statistically significant difference was observed with comparing different treated Ni groups (*p* > 0.05).

Similar results were observed when considering the relative comparison (TST % BL) between treated Ni and control groups. Thus, Ni induced mean average decrease of 14%, 15%, and 20% in males, and of 9%, 7%, and 17% in females, at doses of 0.25, 0.5, and 1 mg/kg, respectively. 

### 3.4. Nickel Effect on Memory

#### 3.4.1. Morris Water Maze

##### Spatial Learning

**Figure 4:**
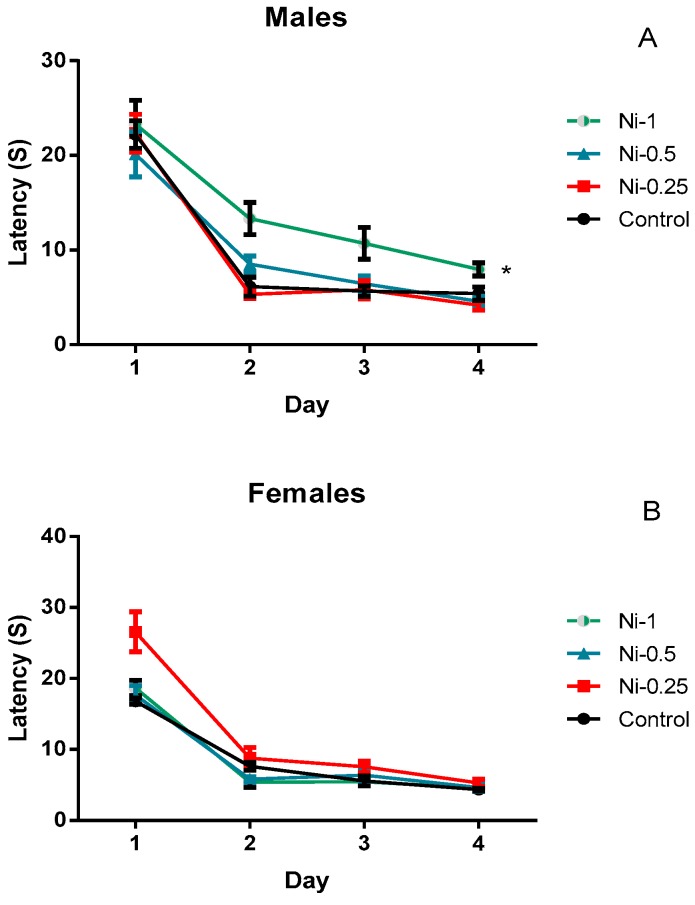
Latency to reach the hidden platform on each of the 4 days of learning phase in the Morris water maze, in male (**A**) and female rats (**B**) after 8 weeks of treatment with 0.9% of NaCl (Control), 0.25 mg/kg (Ni-0.25), 0.5 mg/kg (Ni-0.5), and 1 mg/Kg (Ni-1) of Ni. Results are represented as mean ± SEM. The significance level is 0.05. * *p* < 0.05.

The daily average latency to escape was analysed as a measure of learning in the water maze task (Figure 4). In males, we observed that the group Ni-1 spent significantly more time in finding the hidden platform compared to control (*p* < 0.05). On the other hand, in females, no significant differences in latency to reach the hidden platform between the treated and control groups (*p* > 0.05). In addition, in both sexes, no statistically significant difference was observed upon comparing different treated Ni groups (*p* > 0.05).

##### Percentage Time Spent in the Correct Quadrant During the Probe Test

**Figure 5:**
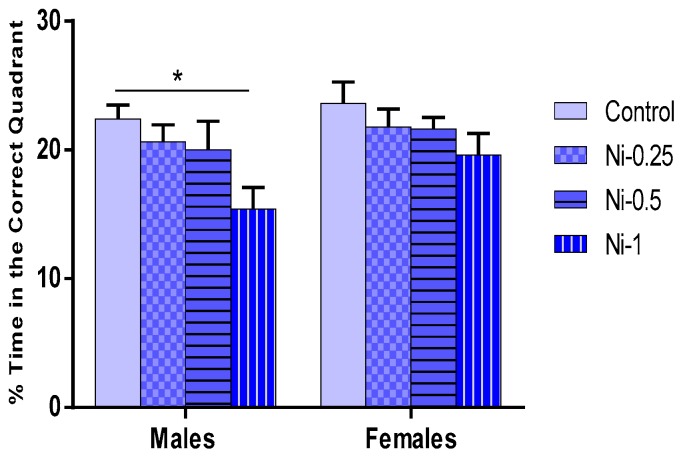
Percentage of time spent in the correct quadrant in the probe test of the Morris water maze expressed as % in male and female rats after 8 weeks of treatment with 0.9% of NaCl (Control), 0.25 mg/kg (Ni-0.25) 0.5 mg/kg (Ni-0.5), and 1 mg/Kg (Ni-1) of Ni. Results are represented as mean ± SEM. The significance level is 0.05. * *p* < 0.05.

Spatial memory was assessed using the percentage time spent in the correct quadrant. The statistical analysis showed that the percentage of time spent in the correct quadrant is significantly affected by Ni treatment (F_(3.32)_ = 4.33, *p* < 0.05) (Figure 5). In males, Ni at dose of 1 mg/kg was associated with a significant decrease of the percentage of time spent in the correct quadrant statistically significant compared to the control group (*p* < 0.05), while at doses of 0.25 and 0.5 mg/kg this metal did not induce any significant change in this parameter (*p* > 0.05). 

In females, Ni did not induce any significant change in this parameter (*p* > 0.05). Indeed, in both sexes, no statistically significant difference was observed with comparing different treated Ni groups (*p* > 0.05). 

The relative comparison (percentage of time spent in the correct quadrant % BL) between treated Ni and control groups shows that Ni induced mean average decrease of 8%, 11%, and 31% in males, and of 8%, 9%, and 17% in females, at doses of 0.25, 0.5, and 1 mg/kg, respectively.

### 3.5. Nickel Effect on Oxidative Stress

#### 3.5.1. Lipid Peroxidation (LPO) in Hippocampus

**Figure 6:**
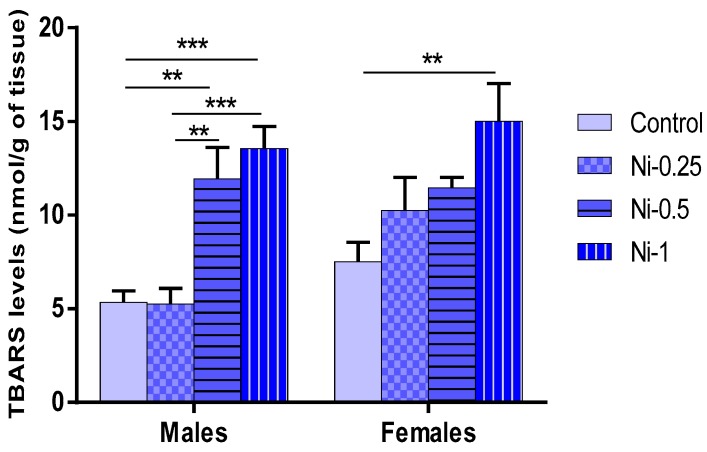
Determination of the lipid peroxidation levels in hippocampus, TBARS levels expressed in nmol/g of tissue in male and female rats after 8 weeks of treatment with 0.9% of NaCl (Control), 0.25 mg/kg (Ni-0.25), 0.5 mg/kg (Ni-0.5), and 1 mg/Kg (Ni-1) of Ni. Results are represented as mean ± SEM. The significance level is 0.05. ** *p* < 0.01, *** *p* < 0.001.

Statistical analysis showed that LPO reflected by TBARS levels was significantly affected the Ni treatment (F_(3.32)_ = 14.92, *p* < 0.001) (Figure 6). The results summarized in Figure 6 showed the following: In males, Ni affects LPO in dose-dependent manner, since at doses of 0.5 and 1 mg/kg, it increases the TBARS levels in rat hippocampus compared with the control group (*p* < 0.01 and *p* < 0.001 respectively), whereas at 0.25 mg/kg Ni was not effective (*p* > 0.05). Indeed, there is a difference statistically significant between Ni-0.25/Ni-0.5 and Ni-0.25/Ni-1 groups (*p* < 0.01 and *p* < 0.001, respectively). No difference was noted between Ni-0.5/Ni-1 groups (*p* > 0.05).

In females, at dose of 1 mg/kg, Ni increases significantly the LPO levels in comparison with control group (*p* < 0.01), while at doses of 0.25 and 0.5 mg/kg this metal did not induce any significant change in this parameter (*p* > 0.05). No statistically significant difference was observed with comparing different treated Ni groups (*p* > 0.05).

Similar results were observed when considering the relative comparison (TBARS % BL) between treated Ni and control groups. Thus, Ni induced mean average increase of 0%, 123%, and 153% in males, and of 36%, 52%, and 100% in females, at doses of 0.25, 0.5, and 1 mg/kg, respectively.

#### 3.5.2. NO Concentrations in Hippocampus

**Figure 7:**
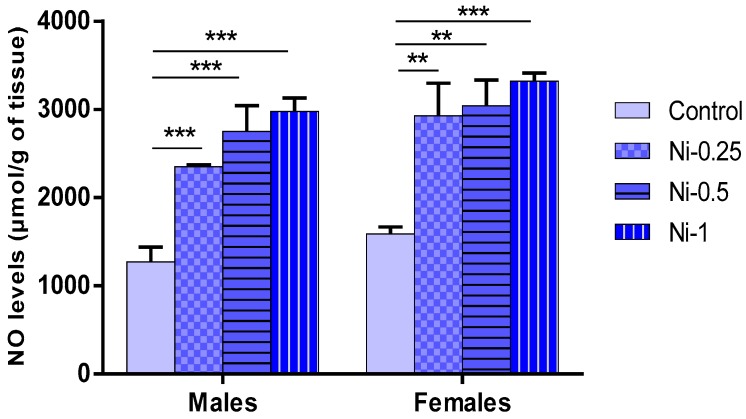
Determination of the nitric oxide (NO) levels in hippocampus, expressed in µmol/g of tissue in male and female rats after 8 weeks of treatment with 0.9% of NaCl (Control), 0.25 mg/kg (Ni-0.25), 0.5 mg/kg (Ni-0.5), and 1 mg/Kg (Ni-1) of Ni. Results are represented as mean ± SEM. The significance level is 0.05. ** *p* < 0.01, *** *p* < 0.001.

This parameter was affected by Ni treatment (F_(3.32)_ = 24.62, *p* < 0.001) (Figure 7). In males, Ni decreases significantly the nitrite/nitrate levels (nitric oxide; NO) in all treated group in comparison with the control group (*p* < 0.001). It induced mean average increase of 84%, 116%, and 133%, at doses of 0.25, 0.5, and 1 mg/kg respectively.

Also in females, Ni affects the NO levels in dose-dependent manner, since at doses of 0.25, 0.5, and 1 mg/kg it increases the NO levels in comparison with the control group (*p* < 0.01, *p* < 0.01, and *p* < 0.001 respectively). The metal induced mean average increase of 84%, 91%, and 108%, at doses of 0.25, 0.5, and 1 mg/kg respectively. In addition, in both sexes, no statistically significant difference was observed with comparing different treated Ni groups (*p* > 0.05).

#### 3.5.3. Superoxide Dismutase (SOD) Activity in Hippocampus

**Figure 8:**
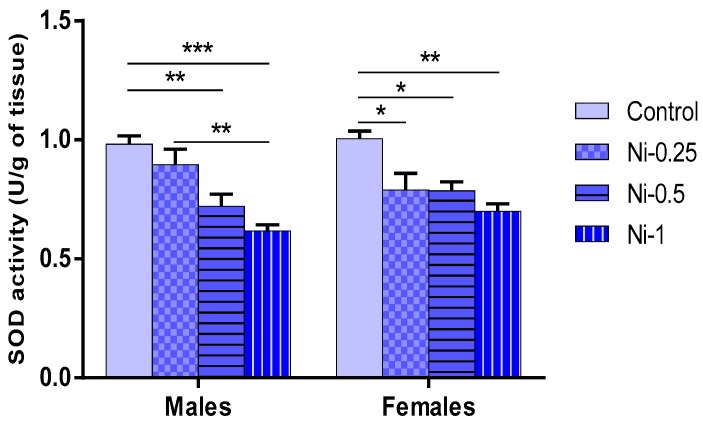
Changes in Superoxide Dismutase (SOD) activity in hippocampus, expressed as U/g of tissue in male and female rats after 8 weeks of treatment with 0.9% of NaCl (Control), 0.25 mg/kg (Ni-0.25), 0.5 mg/kg (Ni-0.5), and 1 mg/Kg (Ni-1) of Ni. Results are represented as mean ± SEM. The significance level is 0.05. * *p* < 0.05, ** *p* < 0.01, *** *p* < 0.001.

SOD activity was affected by treatment (F_(3.32)_ = 19.45, *p* < 0.001), but no effect of sex (F_(1.32)_ = 0.25, *p* > 0.05) was noted. The results summarized in Figure 8 showed the following: In males, Ni affects SOD activity in dose-dependent manner, since at doses of 0.5 and 1 mg/kg, it decreases the SOD activity in rat hippocampus compared with the control group (*p* < 0.01 and *p* < 0.001 respectively), whereas at 0.25 mg/kg Ni was not effective (*p* > 0.05). Indeed, there is a difference statistically significant between Ni-0.25/Ni-1 groups (*p* < 0.01). No difference was noted between Ni-0.25/Ni-0.5 and Ni-0.5/Ni-1 groups (*p* > 0.05).

In females, Ni affects SOD activity at doses of 0.25, 0.5 and 1 mg/kg since it decreases the SOD activity in comparison with the control group (Cont/Ni-0.25: *p* < 0.05, Cont/Ni-0.5: *p* < 0.05, and Cont/Ni-1: *p* < 0.01 respectively). No statistically significant difference was observed with comparing different treated Ni groups (*p* > 0.05). 

The relative comparison (SOD % BL) between treated Ni and control groups shows that Ni induced mean average decrease of 9%, 27%, and 37% in males, and of 22%, 22% and 30% in females, at doses of 0.25, 0.5, and 1 mg/kg, respectively.

#### 3.5.4. Catalase (CAT) Activity in Hippocampus 

**Figure 9:**
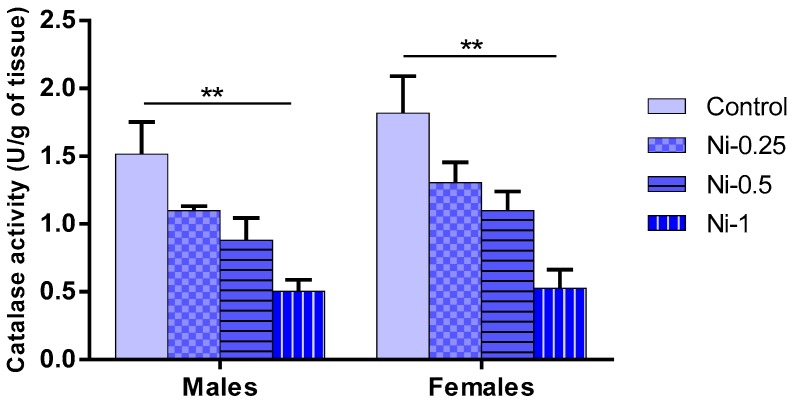
Changes in catalase (CAT) activity in hippocampus, expressed as U/g of hippocampal tissue in male and female rats after 8 weeks of treatment with 0.9% of NaCl (Control), 0.25 mg/kg (Ni-0.25), 0.5 mg/kg (Ni-0.5), and 1 mg/Kg (Ni-1) of Ni. Results are represented as mean ± SEM. The significance level is 0.05. ** *p* < 0.01.

CAT activity was significantly affected by Ni treatment (F_(3.32)_ = 16.72, *p* < 0.001) (Figure 9), but not by sex factor (F_(1.32)_ = 2.45, *p* > 0.05). No interaction was found between treatment and sex F_(3.32)_ = 0.24, *p* > 0.05).

In males and females, Ni at dose of 1 mg/kg was associated with a significant decrease of CAT activity in hippocampus compared with the control animals (*p* < 0.01). In addition, no statistically significant difference was observed with comparing different treated Ni groups (*p* > 0.05).

The relative comparison (CAT % BL) between treated Ni and control groups shows that Ni induced mean average decrease of 28%, 42%, and 67% in males, and of 29%, 40%, and 72% in females, at doses of 0.25, 0.5, and 1 mg/kg, respectively.

## 4. Discussion

The main objective of this study was to determine the effects of chronic exposure to Ni on animal behavior, in particular on affective, cognitive disorders and on levels of oxidative stress. The assessment of anxiety-like and depression-like behaviors is based on the use of validated OFT, EPM, and FST behavioral tests, while spatial learning and memory have been evaluated using the Morris water maze test. Oxidative stress has been determined by measurement of NO, SOD, CAT, and TBARS directly associated with LPO. 

The present study showed that Ni, administered chronically, exerts an anxiogenic effect in rats. Ni decreases the TCA and NRC parameters in the OFT, and TOA and EOA parameters in EPM without modifying the locomotor activity. Our work also showed that in FST, Ni caused an increase in TIM and a decrease in TST in males and females, highlighting the depressant effect of metal. This result was in agreement with the observation of Kahloula et al., (2014), which reported in rats during the development period after oral administration of Ni [18]. Compared with this study, our work has the advantage of administering very small amounts of Ni in a chronic way over a long period of the time and of obtaining effect at lower doses, since the Ni efficiency appears from 0.25 mg/kg. 

Considering the changes in neurochemistry often correlate with behavioral disturbance [17], the increased depressant and anxiogenic effects may be due to alteration in synaptic transmission. After reaching the brain through failures of BBB or via the olfactory pathway [8,9,10], Ni accumulates in the whole brain including cerebral cortex [8,11], leading consequently to a disrupts neurotransmitters [7,16,20,21] then affecting in the long-term, synaptic function and behavior. As low serotonin (5HT) levels and some of the critical brain amines are correlated with depression and anxiety [49], it is possible that the neurotoxic effects of Ni occurs on these neurotransmitters [16]. In this direction, the decrease of 5HT, dopamine, and noradrenaline levels in cerebral cortex and basal ganglia following administration of Ni has already been demonstrated [16]. The inhibition of 5HT biosynthesis by suppressing tryptophan hydroxylase and reducing 5HT receptor gene expression [50] and changes in the expression of dopamine-related genes were also provoked by Ni in vitro [51,52]. It is also possible that the effect of Ni on neurotransmitters concentration was due to decrease of Na^+^,K^+^-ATPase activity [53], since it is implicated in neural excitability, metabolic energy production, as well as in the uptake and release of catecholamines [54] and serotonin [55]. The diminution of brain Na^+^,K^+^-ATPase activity could be resulted from the formation of Ni-ATPase complexes through SH group of enzyme and/or increased oxidative stress [56].

In this study, we also reported that learning and memory were affected by Ni. Especially in males, Ni exposure impaired spatial learning performance, a higher latency was observed in rats exposed to this metal at dose of 1 mg/kg. A deficit of memorization was also shown in Ni-1 group, explained by a significant decrease in the time spent in the quadrant where platform it was localized during the Probe Test. These results are consistent with recent observation showing that Ni caused an impairment in spatial learning and memory in the MWM [18]. Also, in Mice, increased latency in MWM, was obtained after acute intoxication with Ni at 5 and 50 mg/kg [11].

This impairment of spatial learning and memory may be due to the disturbance of the hippocampal circuit and its vast connections [57]; the hippocampus especially being indispensable in the integration of spatial information. Animals that have undergone hippocampal lesions have poor performance in MWM [58], a structure known to undergo morphological changes in rats exposed to heavy metals [59,60]. Therefore, the memory deficit observed in our study might be related to alteration in some cellular and transmitters in the hippocampus and other brain areas. In this sense, the effects of Ni on the cholinergic and glutamatergic system, which plays a crucial role in the process of learning and memory [61], could help to explain the disturbances of this process. Since acetylcholinestrase (AChE) is an enzyme responsible for hydrolyzing acetylcholine, it would be the target of Ni [62,63]. Indeed, the decrease AChE activity in brain of rats submitted to 20 mg/kg/day Ni for 20 days was already reported [56].

On the other hand, Ni affects the function of several different neuronal ionic channels with significant specificity [64,65,66,67], in particular, it modifies the behavior of the *N*-methyl-d-aspartate subtype of glutamate receptor (NR) [68,69], a ligand-gated ion channel that plays a key role in CNS development, synaptic plasticity, learning, and memory [70,71]. Excessive activation of ionotropic glutamate receptors causes a degeneration called excitotoxicity, involved in neurological diseases, stroke, head trauma, and epilepsy. Generally, excitotoxicity is mainly caused by elevated influx of Ca through the Ca-permeable *N*-methyl-d-aspartate (NMDA) subtype of glutamate receptor (NR) [72]. It has been demonstrated that Ni_2_^+^ exerts multiple and complex effects on NR currents [69], which are largely dependent on the subunit (NR2A or NR2B) present in the receptor, it induced potentiation of NR2B-containing receptors, subsequently aggravating the neuronal damage, and the opposite for NR2A-containing receptors which were inhibited by Ni_2_^+^ [68,69,73].

Another explication of Ni action on affective and cognitive disorders was found in the results of examination of same parameters in hippocampus reported in this study. We demonstrate that Ni exposure increased LPO and NO levels, accompanied by a significant decrease in SOD and CAT activities in the hippocampus in male and female rats. These results are in agreement with several recent studies showing an increase in LPO levels with a significant decrease in SOD and CAT activities in the brain rats following Ni administration, suggesting an elevation of oxidative stress [56,74].

It is well known that SOD and CAT constitute mutually a supportive team of defense against free radicals. The decrease in the activities of antioxidant enzymes observed in our study might be due to direct binding of Ni with sulfhydryl groups of enzymes and oxidative modifications of amino acid chains, which alters the enzyme structure and leads to inactivation or decreased activity of enzyme [75,76]. Consequently, the decreased activities of brain antioxidant systems intimate the accumulation of free radicals and increased level of LPO, which increase the oxidative threat in tissue [56]. 

Apparently, Ni increased NO formation, and this event can be one of the main reasons for the heavy metal’s toxic effects. In the study of Guan, it has been demonstrated that the generation of NO was stimulated by Ni [77]. Generally, NO is produced at the cellular level from arginine and oxygen. This reaction is catalyzed by an enzyme: NO synthase (NOS). In addition, NO reacts with the superoxide ion O_2_^−^ to form the ion peroxynitrite ONOO^−^, a very reactive toxic molecule involved in neurodegenerative pathologies. It acts as a powerful oxidant capable of modifying the functioning of proteins, nucleic acids (DNA oxidation) and lipids (LPO) [78,79], which might explain the increase in LPO observed in our study. Once the LPO is provoked, it’s followed by a structural change in biological membranes [80,81,82] or other lipid-containing elements [83,84] causing impaired membrane fluidity and inactivation of several membrane-bound enzymes [85], which provoke a cell death [86], leading to many degenerative illnesses in the central nervous system, as well as psychiatric disturbances [87]. Also, numerous recent studies have shown an association between the disruption of behavioral functioning and high levels in LPO in the hippocampus [34,88,89].

Certain diseases are associated with oxidative stress disturbances such as neuropsychiatric diseases including schizophrenia and some forms of behavior, such as aggressiveness, depression, and anxiety, and also to deterioration of short-term spatial memory [23,28,29,30,31]. Our hypothesis is that behavioral dysfunction observed in the present study (depression-like, anxiety-like, and memory deficit) after Ni administration, might be linked to an increase in oxidative stress in the hippocampus and other brain areas. 

With regard to affective disorder, the major depression and an impaired neuroplasticity mechanisms in the hippocampus show possible associations [90]. It is well known that the major depression was associated with structural brain changes such as a loss of dendritic spines and synapses, as well as reduced dendritic arborisation, together with diminished glial cells in the hippocampus [90]. Also, alterations in hippocampal neurogenesis, in particular, the reduced hippocampal volume have been reported to be linked with the onset and persistence of depressive symptoms [91,92,93]. In this sense, some currently used antidepressants are able to inhibit or reverse hippocampal atrophy associated with major depression [94]. Additionally, central monoamines is associated with a modulation of hippocampal progenitor proliferation and cell survival, the 5-HT, dopaminergic, and norepinephrinergic systems are also strongly implicated in hippocampal neurogenesis [95]. All these data clearly show the link between the hippocampus and affective disorders, suggesting that the symptoms of anxiety-like and depression-like behavior observed in our study may be due to the alteration of the hippocampus function caused by oxidative stress following Ni administration. Oxidative stress could cause death of 5 HT or other neurotransmitter neurons and consequently a decrease in 5 HT brain levels. A decrease in levels of 5-HT, dopamine, and noradrenaline in all regions of the brain has been shown after exposure to Ni [16]. In addition, at the same time point at which anxiety-like and depression-like behaviors were observed, the increase of NO levels was found in the hippocampus of the rat. In this sense, the involvement of NO in the anxiety-related behavior was investigated in both rats and mice by the use of NOS inhibitors and NO donors [96]. The administration of NOS inhibitors showed an anxiolytic-like profile, whereas NO donors induced an anxiogenic-like effect in the EPM [97,98,99,100,101]. Also, It has been suggested that NO is involved in the pathophysiology of major depression [102,103]. Concerning the memory process, the same mechanism of cellular death would be at the origin of neuronal degeneration, especially those releasing the Acetylcholine and Glutamate, causing a decrease in the release of the neurotransmitters. An association between impairment of learning and decreased brain cholinergic activity has well established [104].

## 5. Conclusions

This study suggests that people living in heavy metal pollution environments and continuous exposure to Ni may eventually lead to behavioral pathologies such as affective and cognitive disorders.
